# Identification and analysis of a key long non‐coding RNAs (lncRNAs)‐associated module reveal functional lncRNAs in cardiac hypertrophy

**DOI:** 10.1111/jcmm.13376

**Published:** 2017-11-20

**Authors:** Jian Zhang, Chenchen Feng, Chao Song, Bo Ai, Xuefeng Bai, Yuejuan Liu, Xuecang Li, Jianmei Zhao, Shengshu Shi, Xin Chen, Xiaojie Su, Chunquan Li

**Affiliations:** ^1^ School of Medical Informatics Harbin Medical University Daqing China; ^2^ Department of Pharmacology Harbin Medical University Daqing China; ^3^ College of Computer Science and Technology Heilongjiang University Harbin Medical University Harbin China; ^4^ College Food and Biological Engineering Jimei University Xiamen China; ^5^ College of Medical Laboratory Science and Technology Harbin Medical University Daqing China

**Keywords:** network analysis, long non‐coding RNAs, cardiac hypertrophy, function prediction, random walk

## Abstract

Cardiac hypertrophy (CH) is a common disease that originates from long‐term heart pressure overload and finally leads to heart failure. Recently, long non‐coding RNAs (lncRNAs) have attracted attention because they have broad and crucial functions in regulating complex biological processes. Some studies had found that lncRNAs play vital roles in complex cardiovascular diseases. However, the function and mechanism of lncRNAs in CH have not been elucidated. In our study, to investigate the potential roles of lncRNAs in CH, the Cardiac Hypertrophy‐associated LncRNAs‐Protein coding genes Network (CHLPN) was constructed by integrating gene microarray re‐annotation and subpathway enrichment analyses. After performing random walking with restart in CHLPN, we predicted 21 significant risk lncRNAs, of which 7 (Kis2, 1700110K17Rik, Gm17501, E330017L17Rik, C630043F03Rik, Gm9866 and Ube4bos1) formed a close module with their co‐expressed protein‐coding genes (PCGs). We found that the module might play crucial roles in the development of CH. In particular, 44 PCGs that were co‐expressed with six lncRNAs were enriched in CH‐related biological processes and pathways. We also found that some lncRNAs participated in the competitive endogenous RNA cross‐talk that might be involved in CH. These results indicate that the functional lncRNAs are related to post‐transcriptional regulation and could shed light on a new molecular diagnostic target of CH.

## Introduction

Cardiac hypertrophy (CH) is compensation for heart pressure overload, which is often related to chronic disease such as hypertension. With the development of molecular biology, more studies have focused on the signalling pathways of cell size expansion and apoptosis; some of which are related to CH, such as the mitogen‐activated protein kinase (MAPK) 1, 2, phosphatidylinositol 3‐kinase/AKT 2 and nuclear factor‐κB 3 pathways. There is evidence that CH had a close relationship with cardiomyocyte metabolism. For instance, Ca^2+^ plays a crucial role in the strictly regulated supply of ATP to meet the energy requirements of the cardiac myofibrils 4. This indicates that CH is closely related to body metabolism and signalling pathway changes.

Current research shows that long non‐coding RNAs (lncRNAs) have become important regulatory factors in development of mammalian, including human heart disease 5. LncRNAs encompass >200 nucleotides, with little or no protein‐coding ability, and are less conserved compared with the protein‐coding genes (PCGs)^6^, ^7^. In addition, their expression pattern in multicellular organisms shows high tissue specificity 8, 9, 10, 11. Many heart disease‐associated lncRNAs have been found in cDNA sequence analysis of humans and mice following the invention of Tiling technology 12. For example, the specific expression of lncRNA Braveheart has been found in human heart and mouse embryonic stem cells. Some studies have demonstrated that lncRNAs play an important role in promoting angiogenesis during embryonic development 13, 14 and are helpful for differentiation of heart valves but dysfunction of lncRNA can lead to myocardial infarction 15. Other research has shown that lncRNA Fendrr is essential for initial cardiac development in mice 16. Recently, many studies have reported that lncRNAs play key roles in murine models of CH 17, 18, 19. Specifically, Viereck *et al*. 18 found that overexpression of lncRNA Chast could lead to CH *in vitro* and *in vivo*. Liu *et al*. 17 found that lncRNA H19 overexpression decreased the size of cardiomyocytes in CH models. These results suggest that lncRNAs play an important role in cardiac development and function 20. However, research about the biological function and mechanism of lncRNAs has only begun, and their exact biological function and regulatory mechanism in CH remain unclear.

RNA sequencing (RNA‐seq) is the technique for detecting RNA expression of all genome scale 21. This technique has identified many lncRNAs by mapping reads to the genome *via* bioinformatics. However, there are few publicly available CH‐related RNA‐seq data due to the high cost of RNA‐seq 22. LncRNA expression can also be detected by gene microarray analysis 23. While most of the expression of lncRNAs is often in low abundance, microarray analysis has a higher sensitivity in detecting low abundance lncRNA expression than RNA‐seq has 11. Expression of 849 ncRNAs in adult mouse brain was identified by re‐annotation of the Allen Brain Atlas probe by Mercer *et al*. 8. Similarly, Pang and others identified >1000 ncRNAs expressed in mammalian CD8+ T cells by microarray probe re‐annotation 24. Liao *et al*. 25 verified the accuracy and consistency of re‐annotated probes of gene microarray data. All the above studies have shown that some of the microarray probes could be used to detect expression of lncRNAs with probe re‐annotation, although the lncRNAs were not detected directly.

**Figure 1:**
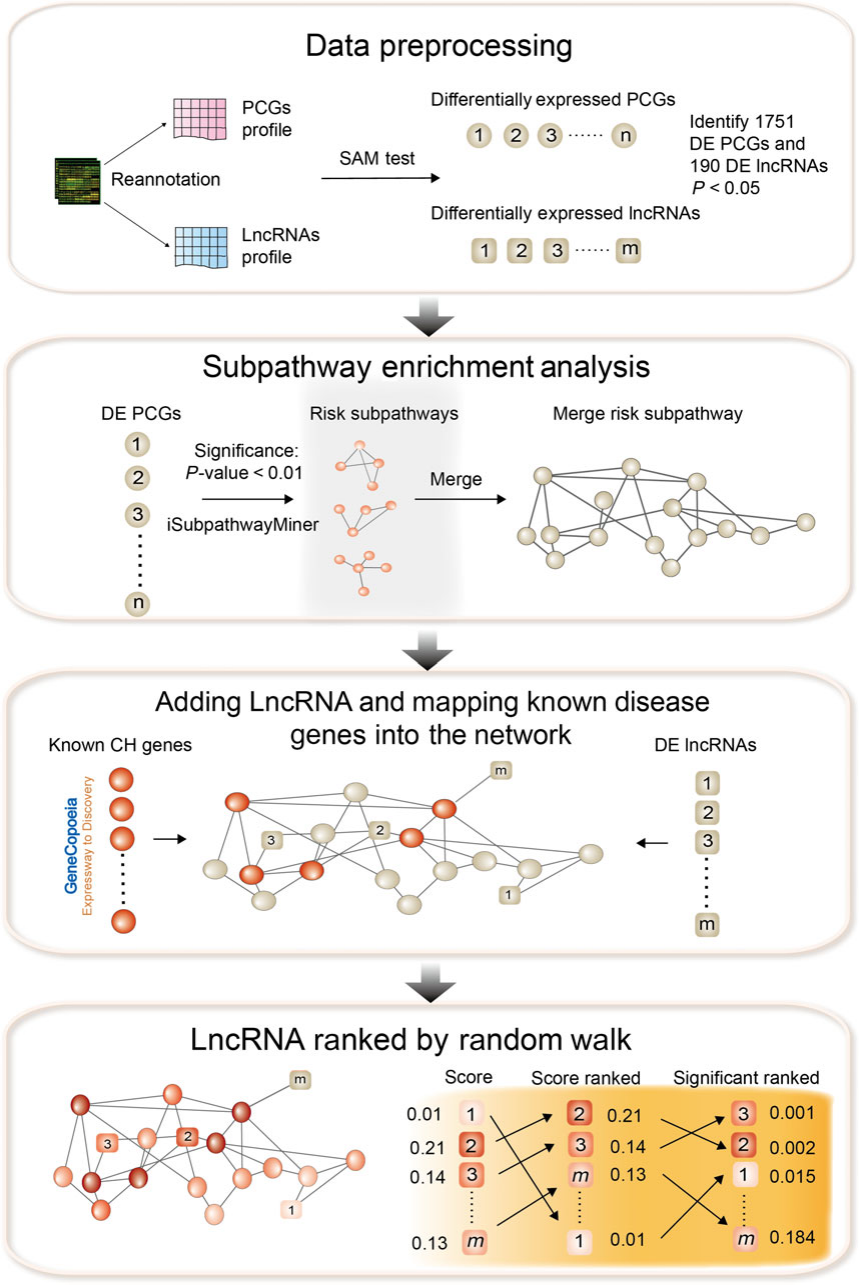
Schematic of the methods. We performed subpathway enrichment for the DEGs and merged the significant subpathways into a network, added the candidate DE lncRNAs that co‐expressed with DEGs into the above network and mapped the disease protein‐coding genes (PCGs) (seed nodes) into the network. We performed the random walking with restart (RWR) method on this network. Finally, we ranked the candidate lncRNAs according to the steady probability of RWR.

In this study, we obtained the expression profile of 16,659 PCGs and 864 lncRNAs from the expression profile data of mice with CH, *via* probe re‐annotation of Affymetrix Mouse Genome 430 2.0 Array (access number of the original profile data is GSE12337 26). We enriched significant subpathways by mapping all differentially expressed PCGs into iSubpathwayMiner, which is an R package that was developed by our group to identify risk subpathways. If PCGs were shared between two subpathways, we merged the subpathways into an undirected network. Furthermore, we added the lncRNAs that were co‐expressed with differentially expressed PCGs into the undirected network. Finally, we generated the Cardiac Hypertrophy‐associated lncRNAs‐PCGs Network (CHLPN), in which nodes represented PCGs and lncRNAs, and edges represented co‐expression of PCGs and lncRNAs or the original regulation relationship in subpathways among diverse PCGs. Moreover, we mapped the known myocardial disease PCGs to the CHLPN and performed the random walking with restart (RWR) method to prioritize CH‐related lncRNAs through comparing their RWR score and significance (Fig. 1). We found that seven lncRNAs (Kis2, 1700110K17Rik, Gm17501, E330017L17Rik, C630043F03Rik, Gm9866 and Ube4bos1) with high scores and significant *P* values formed a close module with their first neighbours in the CHLPN. We then performed hierarchical clustering, gene ontology enrichment analysis and pathway enrichment analysis of the genes in the module. We also identified the competitive endogenous relationships between lncRNAs and PCGs in the module. The seven lncRNAs have a potential function related to CH through directly or indirectly interacting with their co‐expressed PCGs.

## Materials and methods

### Gene expression data

The expression data in this study were downloaded from Gene Expression Omnibus(GEO, https://www.ncbi.nlm.nih.gov/geo/) with accession number GSE12337 26, whose corresponding organism was the mouse, with a total of 16 samples. From these samples, we used four wild normal phenotype and four wild disease data. Wild‐type mice were sham‐operated or subjected to TAC for 28 days, with their left ventricular gene expression profile detected.

### Biological pathways data

Biological pathways were obtained from the KEGG PATHWAY database. Three hundred and forty‐three KEGG pathways were obtained, including 152 metabolic and 191 non‐metabolic pathways. We used the R package SubpathwayMiner to reconstruct all pathways graphically 27. This type of reconstruction retained the raw information of the pathways, particularly the structures, and provided detailed and reliable information for analysing the CH topological properties underlying these biological pathways.

### Probe re‐annotation

We downloaded PCG and lncRNA transcript sequences from Gencode V19 28 and corresponding probe sequences from Affymetrix. We aligned the probe sequences to PCG and lncRNA transcript sequences by sequence alignment tool BLASTn 25, 29. We filtered the probes according to the following rules: (1) keep the probes that exactly matched with transcripts, including PCG and lncRNA transcripts; (2) remove the probes kept in Step 1 that matched lncRNA and PCG transcripts simultaneously; (3) remove the probes kept in Step 2 that matched with multiple lncRNA or PCG transcripts; and (4) each lncRNA or PCG kept in Step 3 can be perfectly matched with at least three probes.

### Identifying differentially expressed lncRNAs and PCGs

We performed log2 transformation of the raw gene expression values. We identified differentially expressed genes in two phenotypes using the SAM function from R package ‘siggenes’. We calculated the fold changes and performed the SAM test for every lncRNA or PCG in two phenotype samples. When fold change was >2 or *P* was <0.05, it was considered statistically significant. The results from the two methods were combined by a union set to generate differentially expressed lncRNAs and PCGs.

### CHLPN construction

In the first step, metabolic and non‐metabolic pathways were divided into a k‐clique subpathway (*k* ≤ 4) by the software package iSubpathwayMiner, which was developed by our research group. We performed subpathway enrichment analysis (*P* < 0.01) for the differentially expressed PCGs (DEGs) to identify the risk subpathways of CH. In the second step, risk subpathways of CH were integrated into a common network based on shared PCG nodes between two subpathways to obtain CHRN, in which nodes and edges were the same in the subpathways. In the third step, the correlation of co‐expression was calculated between differentially expressed PCGs and lncRNAs using the Pearson correlation coefficient (*R* > 0.8). In the fourth step, CHLPN was reconstructed by adding the potential disease‐related lncRNAs generated in the third step into it, creating a new edge between the correlative lncRNAs and PCGs.

### RWR to prioritize lncRNAs

Two hundred and sixty‐three human PCGs associated with cardiomegaly/myocardial disease/ventricular disease were obtained from FULENGEN database (http://www.fulengen.com/product/search/disease/). We converted human orthologous PCGs into mice using Ensembl Biomart tools. They were mapped to CHLPN, as seed nodes for RWR to prioritize lncRNAs related to CH.

A random walk in network is defined as an iterative walker's transition from a certain node to a randomly selected neighbour that started at a source node for given (*e.g*. ‘PCG A’ associated with disease). The random walk that we applied had the capacity of restart with probability *r* in every time step at node PCG A. The RWR was defined as:

$$p^{t+1}=\left(1-r\right)Wp^{t}+rp^{0}$$

where *W* represents the column‐normalized adjacency matrix of the network, *p* ^*t*^ is a vector with size equivalent to the number of nodes in the network, and the *i*‐th element holds the probability of being at node *i* at time step *t*.

In our application, the initial probability vector *p* ^0^ was constructed such that *1* was assigned to the nodes representing known PCGs associated with disease, and other nodes with *0*. We believe that the role of PCGs associated with disease is equivalent in the network. Vector *p* is in the steady state at time step *t*, where *t* approaches infinity as a limit. The iteration is finished when the change between *p* ^*t*^ and *p* ^*t+*1^ falls below 10^−10^.

A random walk algorithm was performed in CHLPN to prioritize lncRNAs related to CH, and we performed statistical significance analysis for the score of every lncRNA. The statistical significance for rejection of the null hypothesis was determined by comparing the scores of lncRNAs in the network following *n* iterations of known PCGs associated with CH shuffling. To maintain the network topological properties, random sampling without replacement was performed when doing the random disturbance, and the degree distribution was guaranteed the same between the selection seed node and the real. In iterations, the times that the score of every lncRNA was higher than the real one was recorded as *m*. The *P* value for every lncRNA was the ratio of *m* and *n*. In this study, *n* was set at 5000 times.

### Prediction of lncRNAs and PCGs by targeting miRNAs

Some studies have shown that some lncRNAs can act as miRNA sponges, namely as ceRNA, and reduce miRNA degradation. Here, we considered that one pair of lncRNA‐PCG shared the common miRNAs was regarded as the ceRNA regulation relation. Firstly, lncRNA‐miRNA interactions were predicted by the popular software: miRanda (www.microrna.org). Briefly, 1915 mature murine miRNA sequences were downloaded from mirBase (www.mirbase.org). The binding sites between lncRNA and miRNA were predicted using an empirical alignment score of 160 and minimum free energy of −20 kcal/mol in miRanda. As for the interaction between PCGs and miRNA, we have downloaded the interaction data between miRNA and PCGs from StarBase 2.0 (starbase.sysu.edu.cn). Then, we could calculate the lncRNA‐PCG interactions based on the shared miRNAs.

## Results

### Construction of CHLPN

After performing sequence alignment between Mouse 430 2.0 array probe sequences from Affymetrix and PCG, lncRNA transcript sequences from Gencode by Blastn tools, we reserved the probe set–RNA pairs that satisfied the filtering rules. In total, 30,344 probeset–RNA pairs were obtained for further research, of which, 29,288 probe sets mapped to PCGs and 1056 mapped to lncRNAs. The PCGs and lncRNAs were represented by Entrez ID.

The differentially expressed transcripts were identified by SAM test. A total of 1226 PCGs and 170 lncRNAs were identified with fold changes >2, and 988 PCGs and 77 lncRNAs were significantly differentially expressed at *P* < 0.05. In total, 1751 differentially expressed PCGs and 190 differentially expressed lncRNAs were obtained by combining the differentially transcripts obtained from two thresholds.

A total of 3029 subpathways from 343 KEGG pathways were obtained (*k* = 4) by applying SubpathwayMiner, which is an R package developed by Li *et al*. Sixty‐five risk subpathways were identified as CH‐related subpathways by subpathway enrichment analysis, which we performed by entering all differentially expressed PCGs into iSubpathwayMiner (*P* < 0.05) (Table S1). We merged all these risk subpathways into a network, and in particular, 655 PCGs in 65 risk subpathways were generated in the cardiac hypertrophy risk subpathway fusion network (CHRN) network, including 7883 edges.

**Figure 2:**
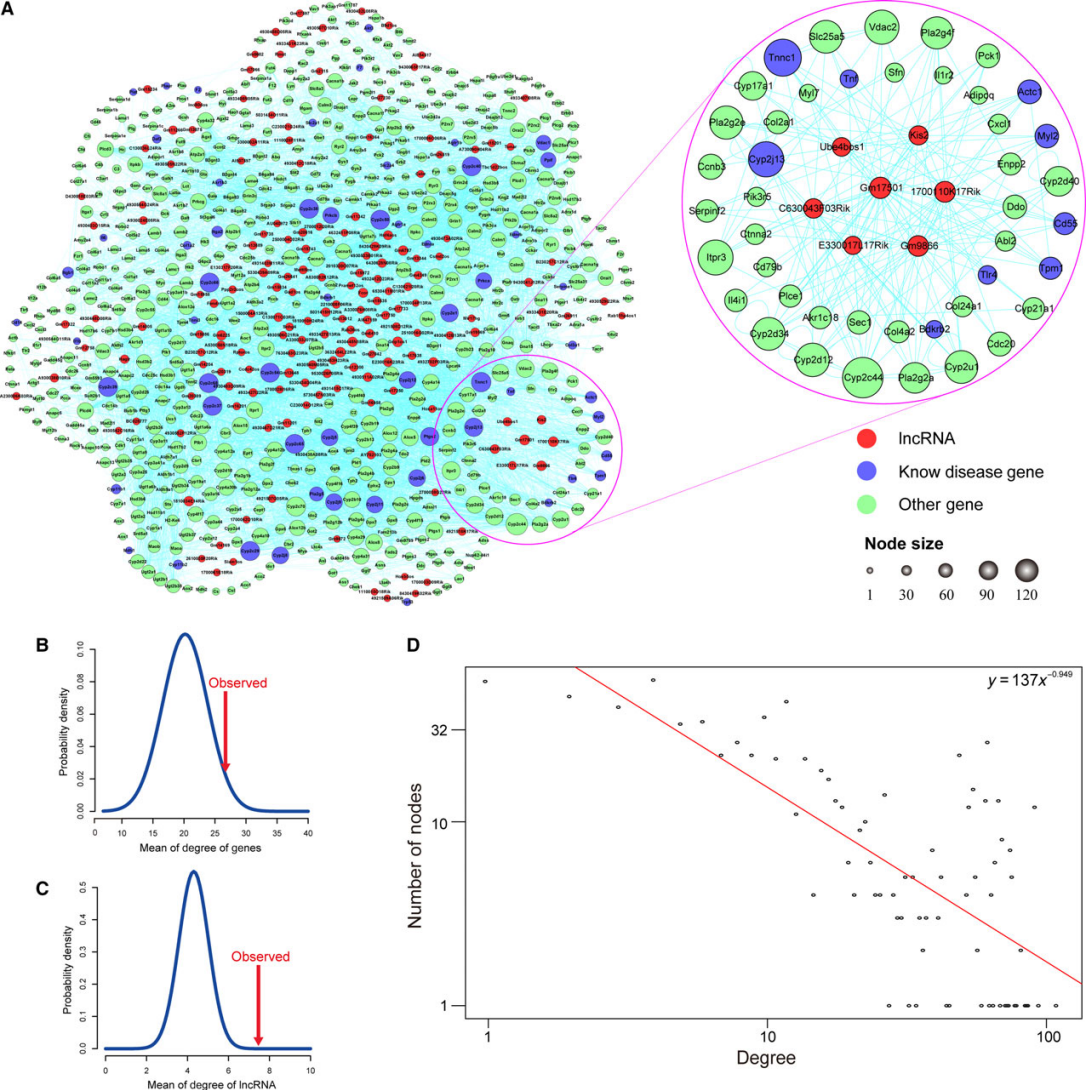
CHLPN network. (**A**) CHLPN networks and key modules. The red, blue and green nodes represent lncRNAs, known disease genes (seed nodes) and other protein‐coding genes (PCGs), respectively. A lncRNA and PCG were connected by an edge if there was a co‐expression relationship between them. The pink circle represents seven risk lncRNAs that ranked in the top 20 by random walk real score and their connected 44 co‐expression PCG nodes, including nine known disease PCGs in CHLPN networks. Node size represented degrees of node (Table S4). (**B**) The blue curve represents the average degree distribution of mRNAs of 1000 times random CHLPN networks; the true CHLPN network's average degree of mRNA was 26.14 (red arrow) and significantly higher than the 1000 times random cases (*P* = 0.026). (**C**) The blue curve represents the average degree distribution of lncRNAs that gained from 1000 times random CHLPN networks, the true CHLPN network's average degree of lncRNA was 7.85 (red arrow) and significantly higher than the 1000 times random cases (*P* = 0). (**D**) The true nodes degree distribution of CHLPN, the degree distribution of all nodes followed the power law distribution approximately with a slope of −0.949 and *R* ^2^ = 0.522.

Co‐expression between differentially expressed PCGs and lncRNAs was calculated by Pearson correlation coefficient. The Pearson correlation coefficient between one pair of differentially expressed PCG and lncRNA was >0.8, which was considered as one co‐expressed pair. LncRNAs were added to CHRN based on co‐expression. The CHLPN was generated, which included 655 PCG nodes, 173 lncRNA nodes and 9241 edges (Fig. 2A). A large component with 824 nodes showed that lncRNAs were closely connected with PCGs, which indicated that lncRNAs and PCGs were intricately related. We reconstructed the lncRNA‐PCG network 1000 times by randomly selecting 1751 PCGs and 190 lncRNAs as the differentially expressed PCGs and lncRNAs. The average degree of lncRNA and PCG nodes in the CHLPN was significantly higher than that in 1000 randomized networks (*P* = 0 and 0.026, respectively) (Fig. 2B and C), indicating that the lncRNAs and PCGs were closely connected at the system level. The degree distribution of all nodes followed the power law distribution approximately with a slope of −0.949 and *R* ^2^ = 0.522 (Fig. 2D). These results revealed that a small number of PCG nodes linked many lncRNA nodes, and a small number of lncRNA nodes that linked many PCG nodes in network act as hubs. In CHLPN, the maximum degree node was Cyp2c44 (degree = 111), the cytochrome P family were crucial members of the arachidonic acid metabolism pathway, and the arachidonic acid metabolism pathway was highly related to the development of CH. Tnnc1 was the second maximum degree node in the CHLPN, which played a key role in cardiac energy supply as a cytosolic Ca^2+^ sensor 30, 31.

### Identification of network key module associated with CH

Two hundred and sixty‐three known CH‐related PCGs were mapped to CHLPN, of which, 59 were found in the network (Table S2, S3). RWR for CHLPN was performed by choosing myocardium‐associated PCGs as seed nodes. This initial score of the seed nodes was set at 1, and we calculated the scores of all lncRNA nodes. To establish whether the lncRNA scores were significantly higher than the random case, we randomly chose 59 of 655 PCG nodes as the seed nodes and performed the RWR 5000 times. As a result, we identified 21 lncRNAs whose scores were significantly higher than that of the random case (*P* < 0.05, Table 1). All these lncRNAs were considered to be risk lncRNAs. We showed that the real scores for risk lncRNAs from RWR were higher than the scores for the non‐risk lncRNAs (*P* = 6.97 × 10^−5^, Wilcoxon rank‐sum test). Among the 21 significant lncRNAs, seven risk lncRNAs were ranked in the top 20 true scores from RWR, namely Kis2, 1700110K17Rik, Gm17501, E330017L17Rik, C630043F03Rik, Gm9866 and Ube4bos1. By mapping these seven lncRNAs into CHLPN, we found that these lncRNAs and their first neighbours formed a close module. Surprisingly, the module contained 44 PCGs and nine of them were known CH‐related PCGs (Fig. 2A). In addition, the average degree of the module was 39.49 and significantly higher than the average degree of other nodes (21.19) (*P* = 3.60 × 10^−10^). This indicated that the cross‐talk between these seven lncRNAs and their related PCGs might play a crucial role in the development of CH.

**Table 1:** List of lncRNA scores significantly higher than random

Entrez ID	Symbol	Score rank	*P* value	Fold change (Log2)
73558	1700110K17Rik	1	8.00E‐04	2.09
100216343	Gm17501	5	0.009	1.91
319894	E330017L17Rik	6	0.0108	−0.61
68285	C630043F03Rik	9	0.0376	1.35
636791	Gm9866	10	0.0232	2.45
751866	Kis2	15	0.0182	1.40
77822	Ube4bos1	19	0.0372	1.14
100504455	Gm15834	24	0.0406	1.47
319830	1500004A13Rik	28	0.0416	0.74
329387	C230014O12Rik	31	0.0102	−1.21
75814	4930467D21Rik	34	0.029	−1.02
78758	4921518K17Rik	38	0.009	1.13
100379612	Gm15886	47	0.02	1.24
100048019	Gm16958	54	0.005	−2.20
100503859	1110015O18Rik	55	0.005	−1.50
75060	4930506C21Rik	63	0.0342	1.43
320879	B230217O12Rik	64	0.0418	1.02
70966	4931415C17Rik	109	0.0436	−1.74
102636239	Gm27042	113	0.0152	1.15
69248	2610035F20Rik	132	0.0108	1.29
100503546	Gm15958	150	0.0204	−1.08

**Figure 3:**
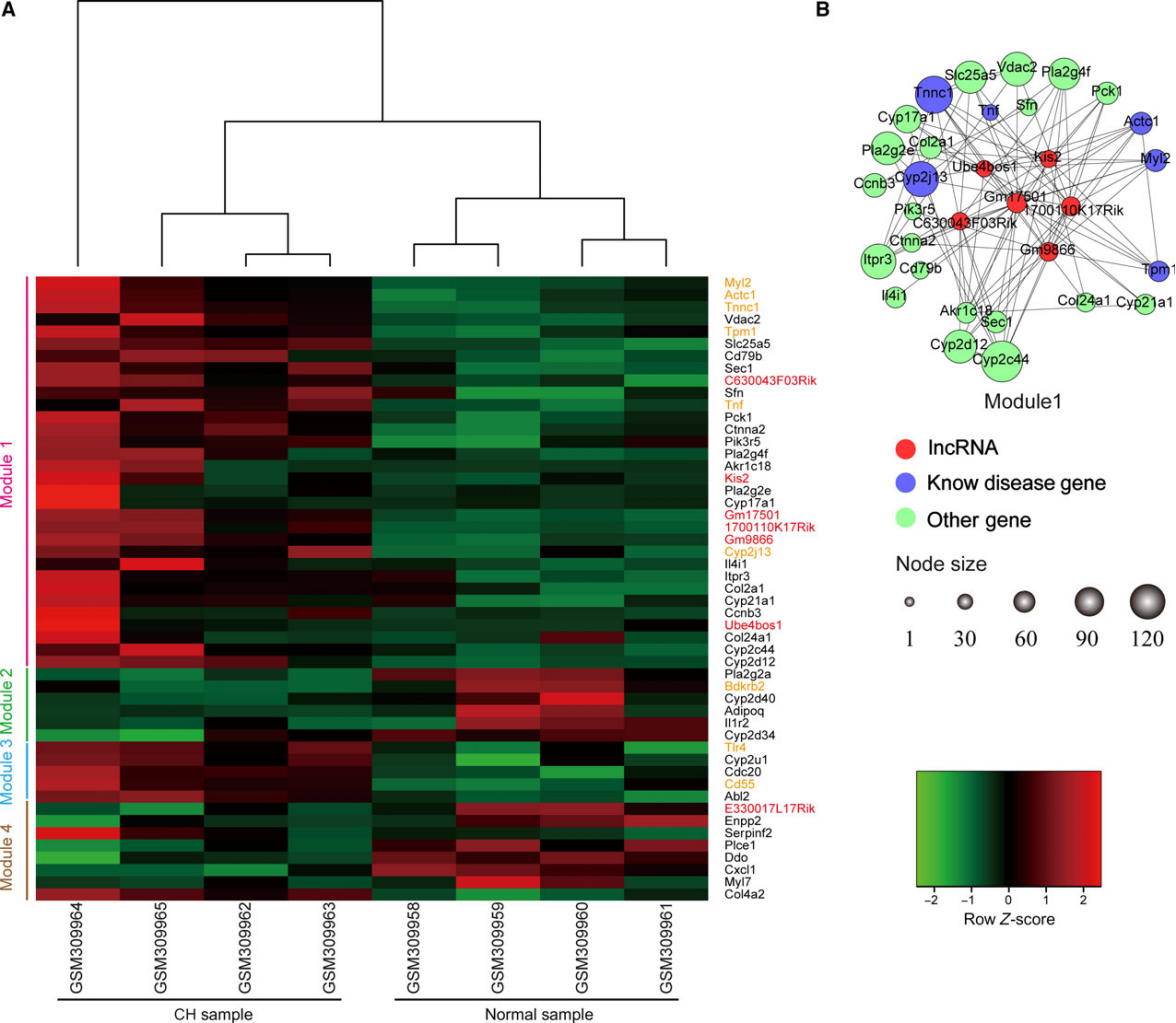
Cluster analyses of key modules associated with cardiac hypertrophy. (**A**) Unsupervised hierarchical clustering of key modules contained lncRNAs and PCGs (rows), samples (columns) is performed, and a heat map was generated. On the right side, the red and orange represent lncRNAs and known disease PCGs, respectively. Seven lncRNAs and 44 PCGs divided the heat map into four groups by hierarchical clustering (submodules 1–4). (**B**) Interaction networks of submodule 1.

For further research of the expression of lncRNAs and PCGs in the module, we performed bidirectional hierarchical clustering. The lncRNAs and PCGs in the module classified the samples into control and disease, suggesting that these lncRNAs and their co‐expressed PCGs possessed potential for diagnosis and therapy of CH. We divided the module into four submodules based on gene expression. The genes in submodules 1 and 3 were up‐regulated and those in submodules 2 and 4 were down‐regulated significantly in the disease samples (Fig. 3A). Specifically, submodule 1 contained six lncRNAs (C630043F03Rik, Kis2, 1700110K17Rik, Gm17501, Gm9866 and Ube4bos1) and 26 PCGs, of which Tnf 32, Cyp2j13 33, Tnnc1, Actc1, Tpm1 30, 31 and Myl2 were known CH‐related PCGs that were higher in the case than control samples with high correlation coefficients (Fig. 3B). Submodule 2, 3 and 4′ structures were loose, but they also contained three known CH‐related PCGs and an lncRNA, which may have been due to the complex biological mechanism involved in development of CH.

### Pathways that regulated by lncRNAs in the key module

**Figure 4:**
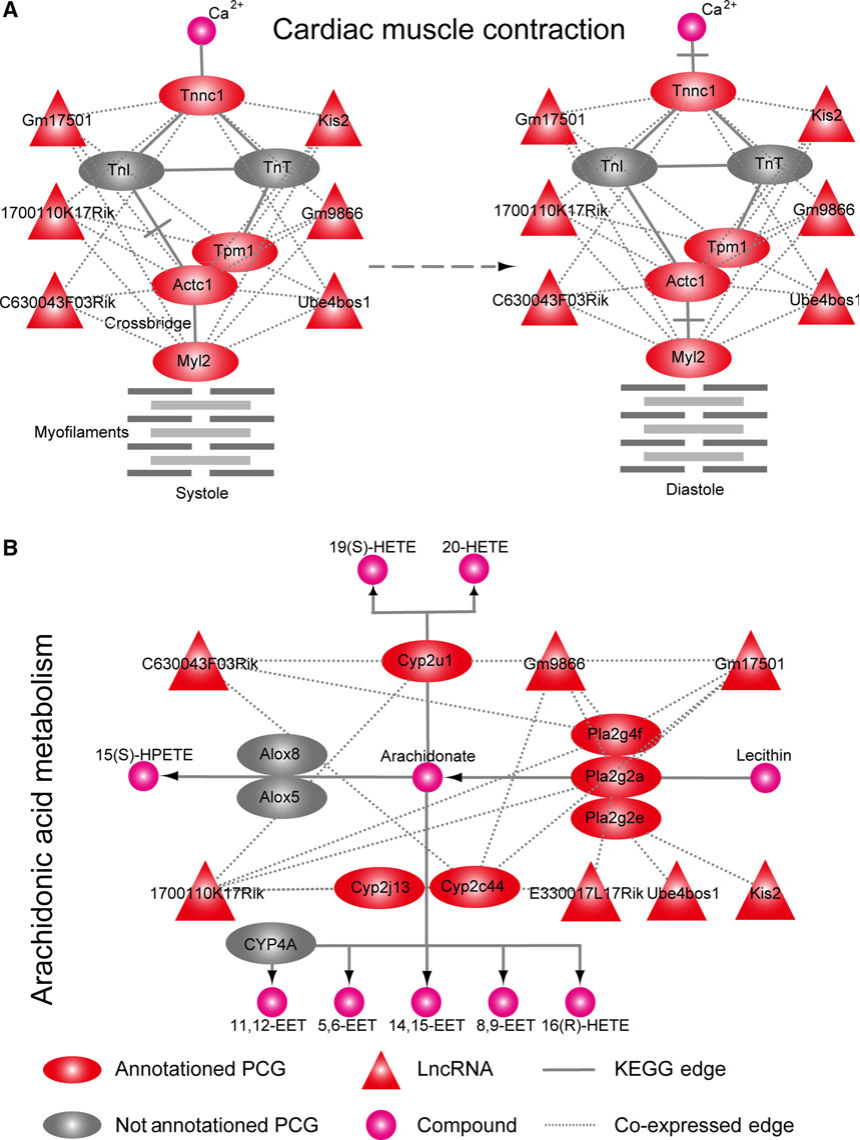
The differentially expressed mRNAs related to lncRNAs were enriched in the pathways. (**A**) Cardiac muscle contraction pathway, (**B**) arachidonic acid metabolism pathway.

The generation and development of diseases are related to changes in biological pathways. The cross‐talk between lncRNAs and PCGs could participate in these changes; thus, it is crucial to understand the mechanism of lncRNA in CH in the pathway dimension. We performed pathway enrichment analysis for the PCGs in the module. There were co‐expression patterns among six lncRNAs (1700110K17Rik, Gm17501, C630043F03Rik, Gm9866, Kis2 and Ube4bos1) and PCGs Tnnc1, Tpm1 and Actc1. Tnnc1, as a cytosolic Ca^2+^ sensor, weakens the inhibitory function of troponin I, causing its release from actin by strengthening the interaction with troponin I 30, 31. Tnnc1 also regulates cardiac systolic or diastolic function by troponin–tropomyosin complex formation with Tpml and Actcl, etc., and is an important part of the cardiac muscle contraction pathway (Fig. 4A) and hypertrophic cardiomyopathy pathway (Fig. S1).

Cyp2u1, Cyp2j13, Pla2g4f, Pla2g2a, Pla2g2e and Cyp2c44 were co‐expressed with seven lncRNAs and significantly enriched in the arachidonic acid metabolism pathway (*P* = 0.000251757) 34, 35, 36 (Fig. 4B). Arachidonic acid was catalysed by Cyp2ul to 20‐hydroxyeicosatetraenoic acid (20‐HETE). There is evidence that 20‐HETE has an adverse effect on the heart and can cause CH 37. We showed that Cyp2u1 was up‐regulated in CH (log2 fold change = 1.29). Phosphatidylcholine was catalysed *via* Pla2g2a to arachidonic acid and formed 5, 6‐, 8, 9‐, 11, 12‐, and 14, 15‐epoxyeicosatrienoic acid, which have a protective effect against CH. However, arachidonic acid may be lowered as result of down‐regulation of Pla2g2e (log2 fold change = −1.74). It was reported that the occurrence of cardiovascular disease is closely related to Pla2g2a 33, 38, 39.

### ceRNA cross‐talk in the key module

lncRNAs can regulate the expression of miRNAs as miRNA sponges, further to regulate the expression of PCGs indirectly and exert functions in the CH. That is to say, lncRNAs exert their function *via* regulation of competing endogenous RNA (ceRNA). Thus, we used the miRanda tools to predict the target miRNAs of the seven lncRNAs. As a result, we found that the 508–530 nucleotide region of the 3′ end of lncRNA Ube4bos1 encompassed miR‐328 binding sites (alignment score = 162, free energy = −32.22 kcal/mol) (Fig. S2A). We also found two other less‐definitive binding sites, with alignment scores of 156 and 150 and free energies of −27.51 and −19.13, respectively (Fig. S2A). Li *et al*. 40 have found that miR‐328 exerts a protective effect against CH through directly reducing sarco/endoplasmic reticulum Ca^2+^‐ATPase SERCA2a expression to activate indirectly the calcineurin/NFATc3 signalling pathway. In our gene expression profile, expression of SERCA2a was up‐regulated in CH samples that compared with control samples (fold change = 1.2). Moreover, the Pearson correlation between SERCA2a and Ube4bos1 was 0.88, which represented a high level of co‐expression between them. There may be a ceRNA between SERCA2a, Ube4bos1 and miR‐328 that could provide a novel therapeutic target of CH. In addition, results showed that the 522–543 sequence of Kis2 was the potential binding region of mmu‐miR‐122‐5p (alignment score = 164, free energy = −24.11 kcal/mol) 41, 42 (Fig. S2B). We also found that mmu‐miR‐122‐5p targeted Cacnb4 in the StarBase database obtained by high‐throughput experiments. The Pearson correlation coefficient between Kis2 and Cacnb4 was 0.87. This indicates that Kis2 and Cacnb4 had positive expression patterns, suggesting that they play as important a role in regulating CH as ceRNA. We ranked the average expression values of all the lncRNAs and PCGs in CH samples. Kis2 ranked in the position of ~9000 and Cacnb4 in ~13,000. The expression level of Kis2 was 2.2 times higher than that of Cacnb4. It is reported that knockdown of p27 increases Kis2 expression in murine lymphoma. Moreover, much evidence suggests that p27 plays an important role in the genesis and development of CH 28, 43, 44, 45. To date, it has not been clarified whether Kis2 works in synergy with p27 in CH.

### Function enrichment of the key module

**Figure 5:**
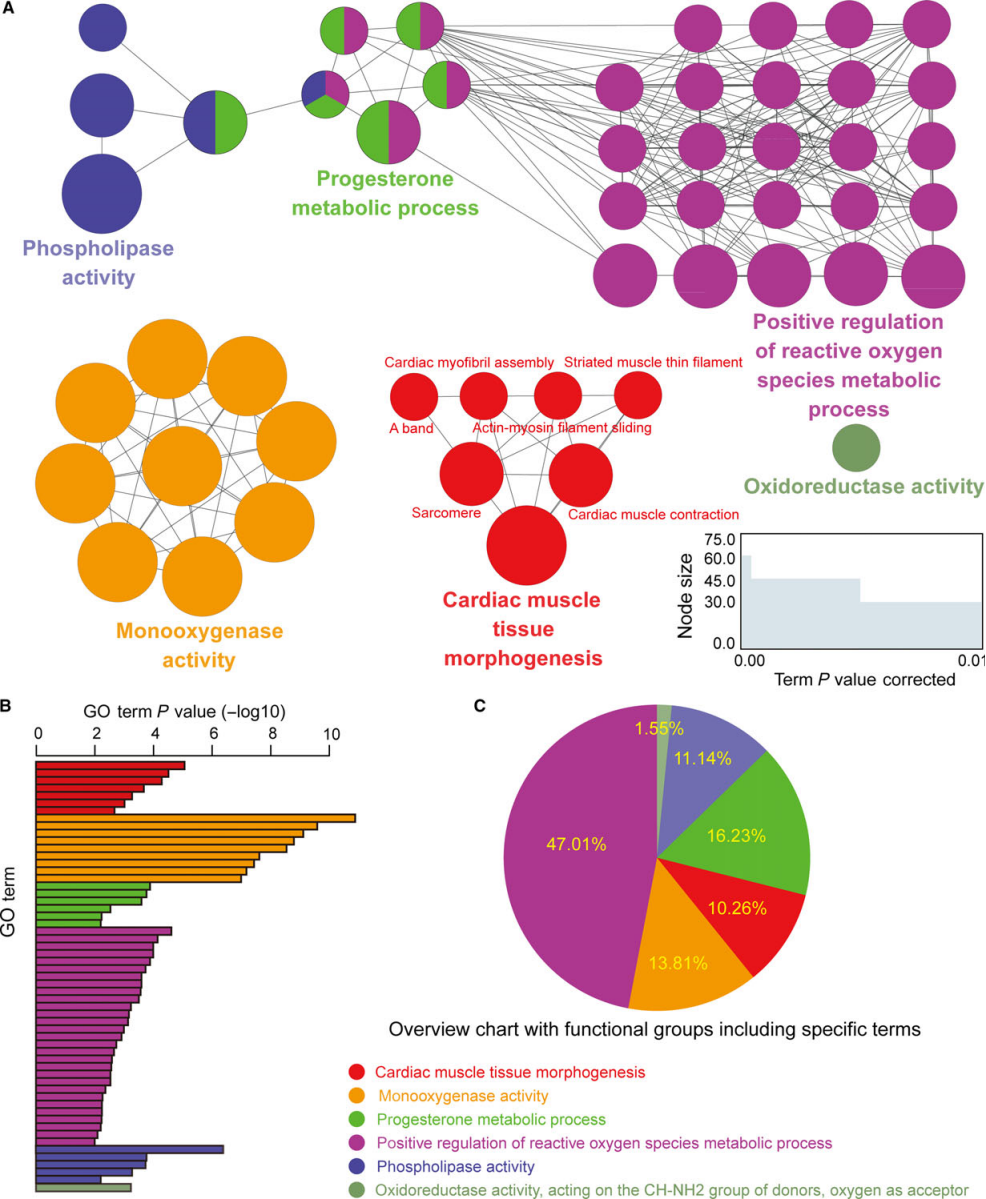
The function enrichment of the key module. Functionally grouped network with terms as nodes were linked based on their κ score (≥0.4), using Cytoscape plug‐in ClueGO. (**A**) Functionally related groups were partially overlapped. The similar GO terms are labelled in the same colour. The size of nodes represented term *P* value corrected with Bonferroni step down. (**B**) GO terms specific for seven lncRNAs and their co‐expressed 44 protein‐coding genes (PCGs). The bars represent the enrichment *P* value of terms (−log10). (**C**) Overview chart of functional groups including specific terms for lncRNAs and their co‐expressed 44 PCGs.

Our hypothesis was that, if the biological function of PCGs is related to the development of CH, and their expression pattern has a positive or negative relationship with lncRNAs, they may be regulated by lncRNAs or may be the downstream or target PCGs of lncRNAs. So, this class of lncRNAs is potentially associated with the development of CH. Using plug‐in ClueGO v2.1.5 in Cytosacpe v3.2.0, 44 co‐expressed PCGs of 7 lncRNAs were enriched for the GO function term. This showed that 11 PCGs were enriched for the GO terms of ‘cardiac muscle tissue morphogenesis’, ‘cardiac muscle contraction’, ‘actin–myosin filament sliding’, ‘cardiac myofibril assembly’, ‘striated muscle thin filament’, and ‘arachidonic acid epoxygenase activity’, all of which were associated with occurrence and development of CH (Fig. 5A). Figure 5B and C shows that the seven lncRNAs and their neighbours in the module were significantly enriched in five categories of GO terms: ‘cardiac muscle tissue morphogenesis’, ‘monooxygenase activity’, ‘progesterone metabolic process’, ‘positive regulation of reactive oxygen species’, ‘metabolic process’ and ‘phospholipase activity’, with enrichment *P* < 0.01.

## Discussion

PCGs and lncRNAs were obtained through probe re‐annotation for the expression profile of CH. We generated a bipartite lncRNAs–PCGs CHLPN using co‐expression analysis and subpathway mining. Seven risk lncRNAs of CH were obtained using the RWR method with known PCGs of cardiac disease acting as seed nodes and formed a close module with their co‐expressed PCGs. We performed cluster, pathway and GO enrichment analysis to investigate the cross‐talk of the key module. We found that the module composed of seven lncRNAs and their co‐expressed PCGs played crucial roles in the origin and development of CH. For example, *via* hierarchical clustering, genes in the module divided the samples into cases and controls, suggesting the important regulatory role of the key module. Through pathway and GO enrichment analysis, some interesting results were discovered. We used the miRanda tools to predict the potential miRNA‐binding site of lncRNAs. We calculated that lncRNA Ube4bos1 has three binding sites for miR‐328, and others have found that overexpression of miR‐328 leads to severe CH 40. MiRanda tools have also predicted that there may be a relationship of ceRNA between Kis2 and Cacnb4 28, 43, 44, 45 *via* mmu‐miR‐122‐5p, suggesting that Kis2 indirectly regulates the expression of Cacnb4 by mmu‐miR‐122‐5p and influences the development of CH. We found that expression level of Kis2 was higher than the expression levels of the PCGs that it potentially regulated. Whether lncRNAs function as sponges when their expression level is higher than that of the PCGs that they regulate is one direction of our future research.

Our study had some limitations. There were insufficient data to form an expression profile of CH at present. The co‐expression between false positive and false negative may appear because there were insufficient samples, which affected evaluating co‐expression of PCGs and lncRNAs. CH‐related 3′ microarray data often focus on testing PCG expression, so fewer lncRNAs were found through probe re‐annotation by microarray analysis. If probe re‐annotation was used in exon microarray analysis, more lncRNAs may be obtained. However, we still found seven lncRNAs and their co‐expressed PCGs, which comprised a close module that might play important modulatory roles in the occurrence and development of CH and offer a new target for diagnosis and treatment.

With the rapid growth of microarray data, we believe that our method could have potential application in CH. In addition, our future research will aim to verify the potential lncRNAs that might play important roles in CH.

## Authors’ contributions

Jian Zhang, Chenchen Feng, Xiaojie Su and Chunquan Li designed the research; Jian Zhang, Chenchen Feng, Chao Song, Bo Ai and Jianmei Zhao performed the research; Jian Zhang, Bo Ai, Xuefeng Bai, Yuejuan Liu, Xuecang Li, Shengshu Shi and Xin Chen analysed the data; Jian Zhang, Chenchen Feng, Chao Song and Xiaojie Su wrote the manuscript. All authors reviewed the manuscript.

## Competing interests

We have no competing interests.

## Supporting information


**Figure S1** Hypertrophic cardiomyopathy pathway.

**Figure S2** Binding alignments of microRNAs targeting predicted lncRNAs. (**A**) Predicted binding alignment of miR‐328‐3P with lncRNA Ube4bos1, 3 binding sites were identified. (**B**) Predicted binding alignment of miR‐122‐5P with lncRNA Kis2.

**Table S1** Risk subpathways of CH (*P* value < 0.05) by applying subpathway enrichment analysis.

**Table S2** Known CH‐related PCGs in CHLPN.

**Table S3** 65 risk sub‐pathways were identified as CH‐related sub‐pathways.

**Table S4** Annotation of all nodes in CHLPN.
