# Supplementary figures and images for: Identification and analysis of a key long non‐coding RNAs (lncRNAs)‐associated module reveal functional lncRNAs in cardiac hypertrophy
Source: J Cell Mol Med. 2017 Nov 20;22(2):892–903. doi: 10.1111/jcmm.13376 (PMC5783834; doi:10.1111/jcmm.13376)

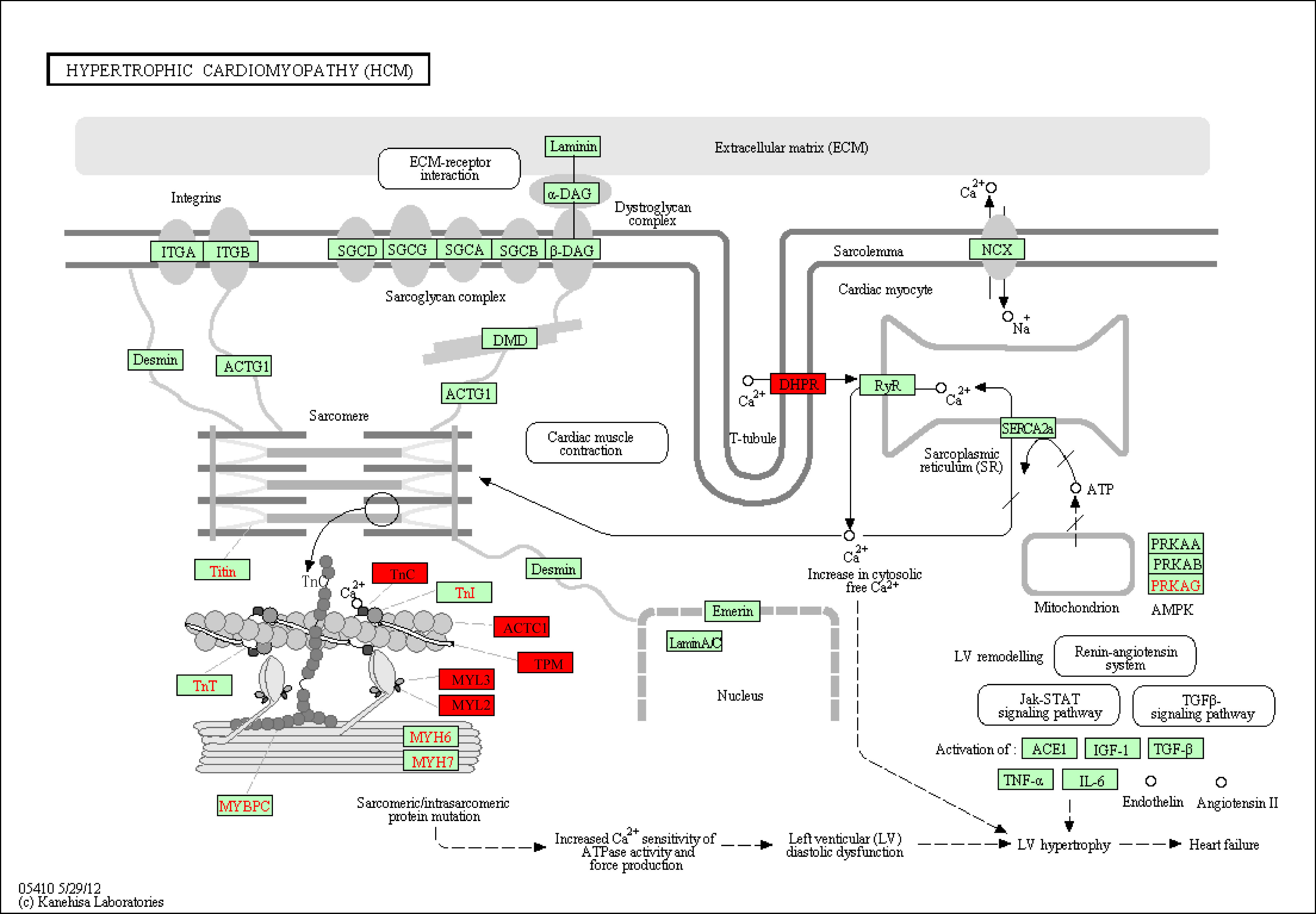

Supplement: Supplementary file 1 — Figure S1 Hypertrophic cardiomyopathy pathway. [file JCMM-22-892-s001.jpg]

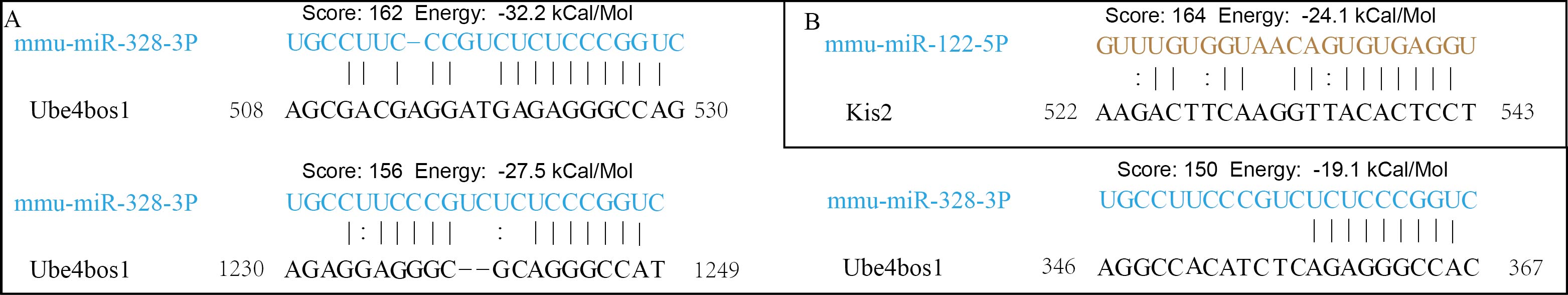

Supplement: Supplementary file 2 — Figure S2 Binding alignments of microRNAs targeting predicted lncRNAs. (A) Predicted binding alignment of miR‐328‐3P with lncRNA Ube4bos1, 3 binding sites were identified. (B) Predicted binding alignment of miR‐122‐5P with lncRNA Kis2. [file JCMM-22-892-s002.jpg]
